# Modeling fashion as an emergent collective behavior of bored individuals

**DOI:** 10.1038/s41598-023-47749-7

**Published:** 2023-11-22

**Authors:** Johannes P.-H. Seiler, Simon Rumpel

**Affiliations:** Institute of Physiology, Focus Program Translational Neurosciences, University Medical Center, Johannes Gutenberg University Mainz, Hanns-Dieter-Hüsch-Weg 19, 55131 Mainz, Germany

**Keywords:** Human behaviour, Cognitive neuroscience, Emotion

## Abstract

Boredom is an aversive mental state that is typically evoked by monotony and drives individuals to seek novel information. Despite this effect on individual behavior, the consequences of boredom for collective behavior remain elusive. Here, we introduce an agent-based model of collective fashion behavior in which simplified agents interact randomly and repeatedly choose alternatives from a circular space of color variants. Agents are endowed with a memory of past experiences and a boredom parameter, promoting avoidance of monotony. Simulating collective color trends with this model captures aspects of real trends observed in fashion magazines. We manipulate the two parameters and observe that the boredom parameter is essential for perpetuating fashion dynamics in our model. Furthermore, highly bored agents lead future population trends, when acting coherently or being highly popular. Taken together, our study illustrates that highly bored individuals can guide collective dynamics of a population to continuously explore different variants of behavior.

## Introduction

Boredom is a common human experience that emerges in many situations of our daily life^1,2^ and across various cultures^3^. Furthermore, boredom has been characterized as an aversive mental state, typically elicited by monotonous and meaningless situations^4,5^. On the level of individual humans, increased boredom proneness has been associated with higher risk-taking^6–9^ and impulsivity^10,11^, but also with positive features such as exploration and creativity^12,13^. Accordingly, boredom has been proposed to serve as a driving force on individual behavior that promotes information-seeking, in order to prevent individuals from repeated exploitation and thus foster exploration^14–16^. Hence, boredom is regarded as a fundamental neurobiological mechanism of individual behavior that may even enfold relevance for non-human species^17,18^.

As an important cognitive driver of individual behavior, boredom could also affect collective behavior which emerges in populations of multiple interacting individuals. However, the concrete and quantitative effects of individual boredom for shaping collective behavior remain elusive.

One particularly vivid example of collective behavior is fashion, which can be characterized by two features: (I) Trends, defined as transient overrepresentations of a certain behavior in a population of agents^19^, e.g. the preference for a certain clothing color, and (II) temporal instability of these trends, meaning that they change and cycle over time. Such ongoing fashion cycles have for instance been reported for colors of clothing^20,21^, length and shape of women’s dresses^22^, but also preferences for baby names^23^, reflecting a variety of behaviors that underlie fashions.

Previous theoretical work, investigating the drivers of trend cycles, suggests that ongoing fashion dynamics strongly depend on the level of conformity of individual behavior with respect to the population^24–26^. Additionally, fashion behavior is qualitatively related to boredom, where increased boredom proneness has been associated with higher frequencies of buying new fashion items^27,28^. This finding on the level of single individuals hints towards a role of boredom that could also affect collective fashion behavior. Nevertheless, a model that describes how a concrete neurocognitive phenomenon, such as boredom, can affect individual conformity and thus shape collective fashion dynamics, has to the best of our knowledge not yet been formulated, hence limiting the current understanding and experimental accessibility of collective trend behavior and its neurobiological basis^29^.

Here, we address and fill this gap by presenting an agent-based framework in order to model fashion trend behavior in a population. Individuals in this population interact, and make sequential decisions on variants from a circular space of alternatives. Individual agents are characterized by only a memory attribute and a boredom attribute, inspired by a recent empirical study on boredom-related behavior^15^. While formulated generally, we specifically apply the model to color trends in fashion and corroborate our simulations by comparing them to a dataset of real color trends in fashion magazines. We then use the model to generate simulations of collective behavior while systematically controlling the degree of boredom in individuals. Analyzing the simulated behavior under various conditions, we observe that boredom has a critical effect on shaping collective trend dynamics.

## Results

### Color trends in fashion magazines

**Figure 1:**
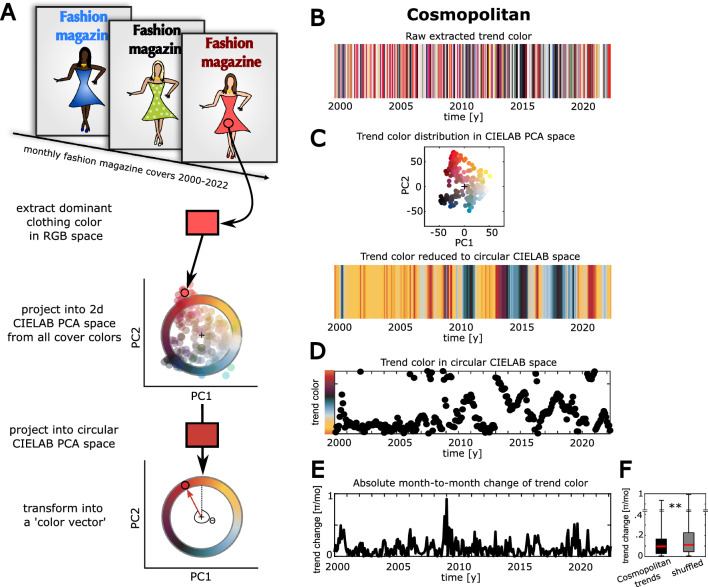
Dynamic color trends in fashion magazines: (**A**) Pipeline of data processing for color trends on fashion magazine covers (time interval 2000–2022). (**B–F**) Color trend dynamics the fashion magazine *Cosmopolitan*: (**B**) Trend colors in RGB color space. (**C**) Upper panel: Trend colors of the respective magazine projected into the plane that explains most variance of all trend colors in CIELAB space. Lower panel: Trend color sequence after projection onto the circular subspace in the plane described above. (**D**) Time-shifted sum over color vectors in a moving bin of 8 months over time. (**E**) Month-to-month change between the trend colors over time (absolute first derivative from the panel above). (**F**) Comparison of the color trend dynamics in E against the dynamics of a color sequence that was randomly shuffled over time (n = 267 trend changes, p-value refers to Wilcoxon rank sum test).

We aimed to model collective trend dynamics in the domain of fashion, focusing our efforts on changing trends of clothing colors. Therefore, we first collected quantitative data on color trends from fashion magazines and characterized them. We analyzed the dominant clothing colors on the monthly covers of the fashion magazine *Cosmopolitan* in the time from 2000 to 2022 and used them as a proxy for fashion trends. In order to quantitatively assess color trends, we analyzed areas featuring the characteristic color of the clothing item shown on the magazine covers, extracted their average color as *RGB* code, and projected this clothing color into a circular subspace of the perceptually even *CIELAB* color spectrum^30^ obtained by principal component analysis (Fig. 1A, see “Methods” section). This procedure reduced the dimensionality of an RGB code to a single scalar, representing the angle between a fixed reference vector and a given color vector in the plane that explained most variance of all colors in the CIELAB space (see “Methods” section).

Consistent with prior work on dynamic fashion trends^21,22^, we observed an ongoing change of trend colors in the Cosmopolitan magazine (Fig. 1B–D), where the circular CIELAB color space was sampled unevenly with dominant clusters of pale yellowish, reddish and blackish colors (Fig. 1C). Interestingly, these color trends showed unsteady dynamics, defined by longer phases of low month-to-month change (i.e. high stability) with short intermittent peaks of high trend change (i.e. low stability) (Fig. 1E). This trend dynamic with varying rates of change was statistically robust against shuffling the trend colors randomly over time (Fig. 1F; Wilcoxon rank sum test: n = 267 trend changes, p = 0.007).

We conducted an analogous analysis of color trends for two independent fashion magazines (*Vogue* and *Harper’s Bazaar*) and found similar distributions of trend colors in the CIELAB space as for the Cosmopolitan (Supplementary Fig. 1D,E). However, we did not find a significant synchrony of trends between the three magazines (Supplementary Fig. 1B,C), indicating a high degree of stochasticity in the cover colors of each particular fashion magazine. Testing the significance of the trend color dynamics of the Vogue and Harper’s Bazaar, we did not find a significant difference from randomly shuffled trend colors (Wilcoxon rank sum test: Vogue p = 0.895, Harper’s Bazaar p = 0.287). On a qualitative level, however, the trend dynamics of Vogue and Harper’s Bazaar matched our previous observations, showing varying rates of trend change over time.

Together, this indicates that color trends in fashion magazines can arise independently from each other and undergo non-random dynamics over time with unstable rates of change.

### Modeling collective behavior with a population of bored individuals

**Figure 2:**
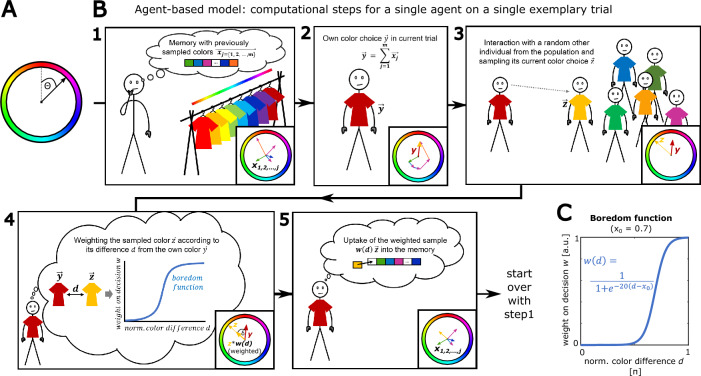
A model of individual boredom and collective fashion behavior: (**A**) Colors in a circular space are represented as a two-dimensional color vectors. The color value is given by the angle that this color vector forms with a fixed reference vector. The hue space is selected as an illustrative example for a circular color space. (**B**) The concept of color vectors is then used in an agent-based model, where single agents integrate multiple sampled colors, form individual decisions for a color variant and randomly interact with other individuals from the population. Each computational step is implemented for each individual from the population. The depicted manikin illustrates the analogy of a subject that iteratively makes fashion-related decisions such as choosing a color for clothing and sampling the colors that other individuals are wearing (see “Methods” section). (**C**) Sigmoid function which is used in the model to implement boredom as novelty-dependent decision-making. The function is constrained in its maximum and skewness, so that the only free parameter defining the boredom of an individual is x_0_, describing the function’s horizontal shift (high x_0_ values reflecting high boredom, low x_0_ values reflecting low boredom). The depicted curve with x_0_ = 0.7 reflects an intermediate level of boredom.

In order to investigate the role of boredom for collective behavior, we devised an agent-based framework to model a population of individuals that repeatedly form decisions for behavioral alternatives from a circular space of variants. We use this framework to model the individuals’ fashion-related choice for clothing colors in a circular space (we emphasize a conceptual congruence with the analyses from Fig. 1, but for illustrative purpose switch to a circular hue color code in our next figures). Colors are expressed and processed as two-dimensional vectors, with the angle between them providing a measure for their dissimilarity (Fig. 2A, “Methods” section).

**Figure 3:**
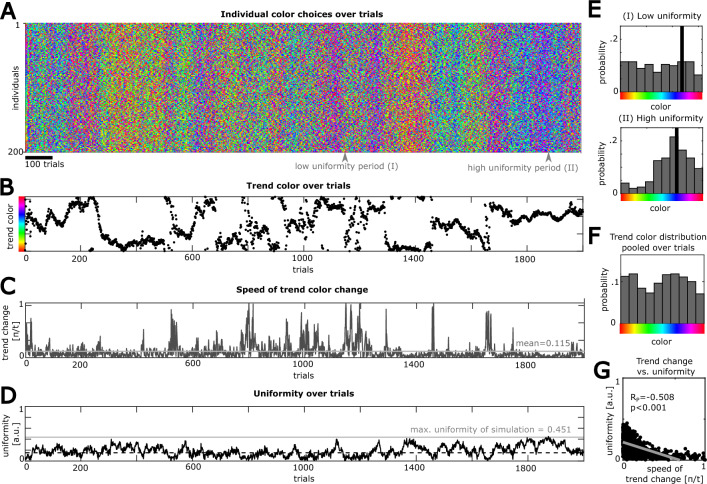
A homogenous population with intermediate boredom exhibits reliable collective fashion trends: (**A**) Color choices over time of all simulated individuals with an intermediate boredom parameter (model parameters: 200 individuals, 2000 trials, memory size m = 12, boredom function shift x_0_ = 0.7). (**B**) Trend color of the population, computed as the summed color vector of all individuals on a given trial, over time. (**C**) Trend change speed, computed as the absolute trial-to-trial difference of the trend color in the population over time. (**D**) Uniformity of the population’s color choices over time, computed as the normalized length of the summed color vector over all individuals on a given trial. The dashed horizontal line indicates chance level uniformity (see Methods). The solid horizontal line indicates the maximal uniformity over all simulated trials. (**E**) Color distributions at two exemplary periods of the simulation (indicated by arrow heads in A, n_i_ = 200 individuals). Upper panel: Low uniformity period with broadly distributed colors across individuals. Lower panel: High uniformity period with unimodal color distribution, matching the current trend color (vertical black bar). (**F**) Distribution of all trend colors of the simulation, where trend colors are evenly distributed across the circular color space (n = 2000 simulated trials). (**G**) Pearson correlation of the population’s uniformity and the population's trend color change (n = 2000 simulated trials).

**Figure 4:**
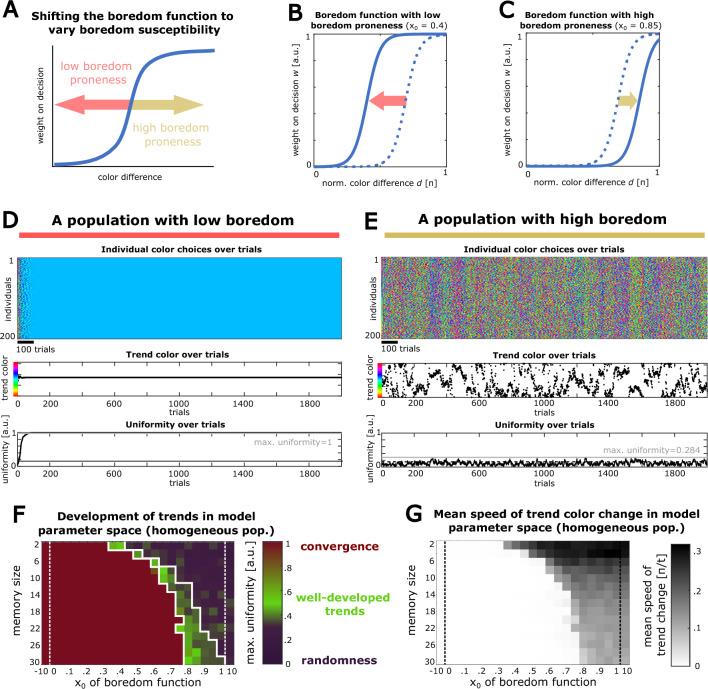
Altering boredom in a homogenous population can critically affect collective trend dynamics: (**A**) Schematic of how changes in the boredom function of the model can be used to implement different degrees of boredom in the population. A right-shift (higher x_0_ parameter) of the boredom function simulates higher boredom susceptibility among agents, whereas a left-shift (lower x_0_ parameter) simulates a lower boredom susceptibility. (**B**) Manipulated boredom function, used to simulate low boredom proneness in the modeled population. (**C**) Equivalent manipulation to simulate increased boredom proneness. (**D**) Simulated trend color dynamics in a population with low boredom proneness (boredom function of B, n_i_ = 200 individuals, memory size m = 12). Upper panel: Individual color choices over trials with converge to a single color. Middle panel: Trend color of the population over time. Lower panel: Uniformity of the population over the simulated trials rising to a maximum of 1. (**E**) Same as D, only for a simulation with a highly bored population (boredom function of C, n_i_ = 200 individuals, memory size m = 12). Here, individuals show a greater mean trial-to-trial change in color trends over time, accompanied by a lower maximal uniformity. (**F**) Matrix of simulations across the space of different combinations of the two model parameters, memory size and boredom parameter x_0_. The color reflects the maximal uniformity of the simulation (simulations with uniformity values between 0.345 and 0.700, representing well-developed collective trends are outlined in white). (**G**) Absolute speed of change in trend colors (mean angle between subsequent trend color vectors in units of pi) over the parameter space for the same simulations as in F.

**Figure 5:**
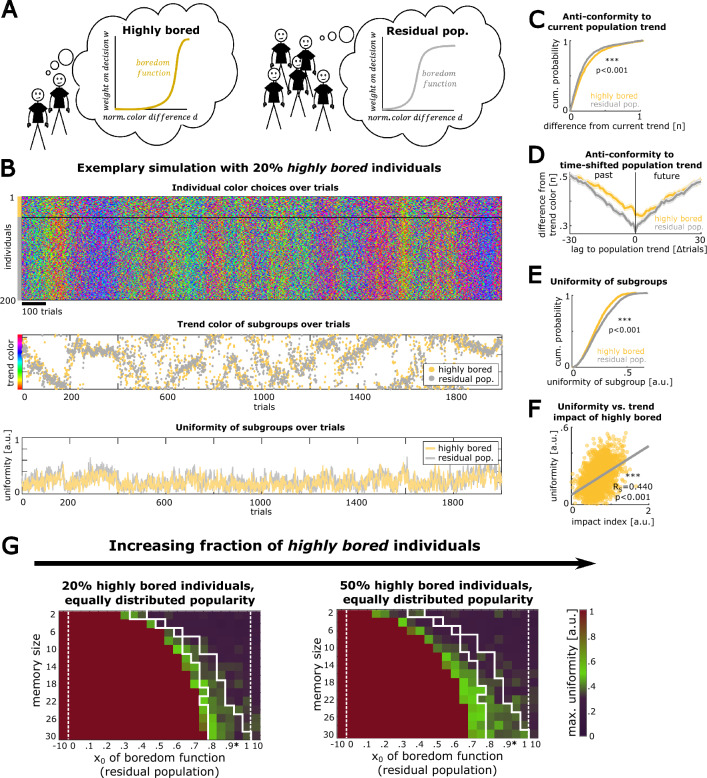
A subpopulation of highly bored individuals affects collective fashion trends: (**A**) Schematic, illustrating that a defined subset of individuals from the population is constrained to have high boredom (boredom function with x_0_ = 0.9). (**B**) Exemplary simulation with a fraction of 20% highly bored individuals (other model parameters: n_i_ = 200 individuals, memory size m = 12, residual boredom x_0_ = 0.65). Upper panel: Individual color choices over trials, grouped according to highly bored (yellow bar) and residual individuals (gray bar). Middle panel: Trend colors of the subpopulations over time. Lower panel: Uniformity of the two subpopulations over time. (**C**) Absolute difference in the subpopulations’ trend colors from the full population's trend color (n = 2000 trials, p value of a Wilcoxon signed-rank test is reported). (**D**) Mean absolute difference between the sequence of subgroup trend colors and the sequence of the full population trend colors, shifted for- and backwards in time. Shaded areas indicate the SEM (n = 2000 trials). (**E**) Uniformity between subgroups (n = 2000 trials, p-value refers to a Wilcoxon signed-rank test). (**F**) Pearson correlation between the uniformity of the highly bored subgroup and its impact on the population trend (see “Methods” section). (**G**) Maximal uniformity of simulations across the parameter space for populations with two fractions of highly bored individuals. The x_0_ value used for highly bored individuals is marked by an asterisk. The white frames are reproduced from Fig. 4F for comparison of the parameter conditions that lead to well pronounced trends with intermediate uniformity in a homogenous population of agents.

The model algorithm implements agents based on the analogy of a human that (i) on each morning has to choose a clothing color for the day, where clothing in all possible colors from the circular space is available (Fig. 2B-1). (ii) The individual has a limited memory of previously experienced colors and decides to dress in the average over all memorized colors (Fig. 2B-2). (iii) Next, as the individual is part of a larger population, it randomly samples the color that another agent is wearing (Fig. 2B-3). (iv) While sampling this other color, the individual compares it to its own current clothing color: If it is very similar to the own color, it is judged as boring, having a low impact on future decisions. However, if it is different from the own color, it is considered as interesting and has a higher impact on following choices (Fig. 2B-4). (v) Lastly, the sampled color is weighted by the degree of boredom it caused, and integrated in the individual’s memory (Fig. 2B-5).

For this model logic, individual agents are characterized by two attributes: First, a *memory* of a given size defining how many trials of the past affect an individual’s current choice. Second, a boredom term (*boredom function*) that defines how the current sample is weighted based on its difference from the current own color, before being transferred to the memory. Motivated by a previous human study^15^, we compute decision weight in the boredom function as a sigmoid of the angular difference between colors, such that low difference (i.e., high boredom) leads to low impact on an individual’s choice and vice versa (Fig. 2C, “Methods” section). In contrast to general frameworks of novelty-seeking which often rely on curiosity-based attractors^31^, our boredom parameter operates in a non-directed manner, facilitating choices which differ from the own current color, hence pushing individuals away from monotony. Shifting the boredom function horizontally along the axis of color difference (implemented by different values of the boredom function’s parameter x_0_) enabled us to simulate individuals with different degrees of boredom proneness (see Methods).

Utilizing this model, we then simulated the color choices in a homogenous population (n_i_ = 200 individuals with memory size m = 12 trials, boredom function shift x_0_ = 0.7, n_t_ = 2000 trials), and tested whether the collective behavior reflects the two characteristic features of fashion dynamics, namely an overrepresentation of a particular color at a given time point (*trend*), that however, is unstable and changes over time. We found that the few model parameters suffice to generate pronounced trends in the population (Fig. 3A,E). Furthermore, the population’s trend color (see “Methods” section) undergoes ongoing changes over trials, sampling the space of possible colors evenly (Fig. 3B,F; Kolmogorov-Smirnoff test for continuous uniform distribution: n = 100 randomly sampled trend colors, p = 0.303). Interestingly, the model also captures the observation from empirical color trends in fashion magazines, namely that the dynamics of trend colors is unsteady, showing longer periods of low trend change with intermittent short periods of high trial-to-trial change (Fig. 3C).

To obtain a metric that captures the coherence of color choices, representing the strength of a trend, we calculated the *uniformity* as the length of the population’s summed color vector at a given time point normalized by the population size (see “Methods” section). We found that uniformity changes over time, reflecting that the homogeneity of color choices undergoes substantial fluctuations (Fig. 3D). During periods of high uniformity, a clear unimodal color trend was observed, whereas during periods of low uniformity, the population shows a vastly even distribution of colors (Fig. 3E). On most trials, however, the uniformity of the simulated population exceeds a chance threshold level obtained by randomly created color vectors (see “Methods” section). Interestingly, when testing the relationship between uniformity and absolute change in the population’s trend color, we observed a remarkable negative correlation (Fig. 3G). This indicates that during an ongoing trend period with low trend color change, the population shows a high degree of uniformity. However, in periods of changing trends, the population diverges and individuals sample different parts of the color space, resulting in reduced uniformity.

### Altering the degree of individual boredom critically affects collective trend behavior

In order to test how the different model parameters affect the simulated collective trend dynamics, we first investigated the effect of population size. Here, we found that large population sizes (in the range of 400–1000 individuals with the above parameter settings) can affect how strongly trends are expressed in a population (Supplementary Fig. 2A,B). The most pronounced fashion dynamic with moderate levels of uniformity was observed in intermediately sized populations (100–300 individuals), so that we conducted the following simulations using a population size of n_i_ = 200 individuals, unless otherwise indicated.

We then investigated, how a manipulation of the population’s level of boredom affects fashion trend dynamics. Therefore, we utilized the aforementioned parameter settings that led to robust and ongoing fashion trend cycles (see Fig. 3), and systematically varied the horizontal shift of the boredom function (parameter x_0_) for all individuals in the population in parallel (Fig. 4A, see “Methods” section). In order to simulate a population with low boredom, we utilized a small x_0_ value, shifting the boredom function to the left (Fig. 4B), whereas for simulating a highly bored population, we utilized a large x_0_ value, shifting the boredom function to the right (Fig. 4C).

Interestingly, we observed that a low level of boredom leads to a rapid convergence of the population’s color choices, resulting in complete uniformity together with a stagnancy of trend dynamics (Fig. 4D). In contrast, when increasing the degree of boredom in a population, color trends become more unsteady and random, reflected by a higher mean change of the population’s trend color and a lower maximal uniformity. This increasing randomness impeded the development of trends per se (Fig. 4E, max. uniformity drops from 0.451 with x_0_ = 0.7 to 0.284 with x_0_ = 0.85 while chance uniformity is 0.153 (see “Methods” section)). A full disruption of any collective trend and levels of uniformity indistinguishable from chance were observed in an extreme simulation with high boredom (x_0_ = 0.9) and a very large (n_i_ = 2000 individuals) population size (Supplementary Fig. 2C). Together, this demonstrates that boredom can play a critical role in navigating collective trend behavior between stagnancy and randomness.

To further characterize collective trend behavior across the parameter space, we systematically tested different combinations of the population’s memory size (m) and boredom (x_0_). As a general read-out of collective trend behavior, we computed the maximal uniformity observed in the respective simulation, since this measure allows reliable identification of convergence or high randomness in collective trend behavior (see “Methods” section). We found that the individual memory size and the boredom parameter x_0_ have opposing effects on collective trend behavior (Fig. 4F): As aforementioned, high boredom counteracts convergence, whereas a large memory size to integrate prior colors promotes convergence. These two antagonistic model parameters, when balanced, allow for functional fashion dynamics, where trends develop reliably but do not converge and can change over time.

As a measure for general turnover of fashion trends in the population, we further analyzed the mean trend color change in the population (Fig. 4G). Here, we observed a higher speed of trend changes predominantly in conditions with low memory size. The population’s degree of boredom also had a promoting, but comparably small effect on the speed of trend change.

### Highly bored individuals prevent collective behavior from convergence to monotony

We next sought to test collective dynamics in a heterogeneous population with different degrees of boredom. For this purpose, we simulated collective trend behavior while constraining a fraction of individuals from the population to be highly bored (implemented by a boredom function parameter of x_0_ = 0.9, Fig. 5A, see “Methods” section).

We observed that a population with 20% highly bored individuals in a balanced model regime (parameters comparable to previous simulations) shows well-developed, ongoing trend dynamics (Fig. 5B). To specifically test the role of these highly bored individuals on population trends, we separately computed the trend color of the highly bored subgroup and the trend color in a random subsample of residual individuals of the same size. We then compared the trend colors from both subgroups to the trend color of the full population. We observed that highly bored individuals differ more strongly from the current population trend, indicating anti-conformist behavior in this subgroup (Fig. 5C, Wilcoxon signed rank test: n = 2000 trials, p < 5.5e−10). Interestingly, when computing the mean difference between the time-shifted full population trend and the trend of the highly bored or residual subgroup, we found that the highly bored subgroup has its difference minimum (i.e. the highest similarity) to population trends in the close future (Fig. 5D, see “Methods” section), indicating avantgarde behavior of highly bored individuals. In contrast, the residual population showed an overall lower difference from the full population trend due to its dominant share in the population, with a minimum at the current trial. Furthermore, the subgroup of highly bored individuals displayed a generally lower uniformity throughout the simulation (Fig. 5E, Wilcoxon signed rank test: n = 2000 trials, p = 1.2e−18). In order to assess the role of highly bored individuals on changes in the population trend, we computed an *impact index* to express the contribution that a single individual has made to drive the change in the population’s trend color on a given trial of the simulation (see “Methods” section). This impact index is based on the frequency of being sampled by other agents and the weight of this sample on their color choices in relation to the trend color. We found that the average impact index in highly bored individuals is positively correlated to the uniformity of the highly bored subgroup (Fig. 5F, n_t_ = 2000 trials, Spearman’s Rho = 0.440, p < e−300). This indicates that the highly bored subpopulation, which in most phases of the simulation shows a comparably low uniformity, is most effective in influencing the population trend when transiently gaining a high degree of uniformity.

To test the role of highly bored individuals in collective trend behavior over a wider parameter space, we again used the maximal uniformity observed in the respective simulation as a general read out. When starting from a homogeneous population and gradually increasing the fraction of highly bored individuals (see “Methods” section), we found that highly bored individuals prevent the convergence of trend behavior in parameter conditions that show convergence for a homogenous population, but also introduced random collective behavior for parameter combinations where clear trends were observed in a homogeneous population (Fig. 5G, Supplementary Fig. 3). This shift in the space of parameter combinations with reliable fashion trend dynamics was not significantly different from a homogeneous population in which the boredom parameter x_0_ was similar to the average boredom in the heterogeneous population (Wilcoxon rank sum test: n = 120 simulation pairs, p = 0.088, see “Methods” section). Together, these observations suggest that the population trend predominantly changes during periods in which a subset of highly bored, anti-conform individuals behave more synchronously than residual individuals, hence safe-guarding the population from convergence and stagnancy.

### Highly bored individuals can drive future population trends

**Figure 6:**
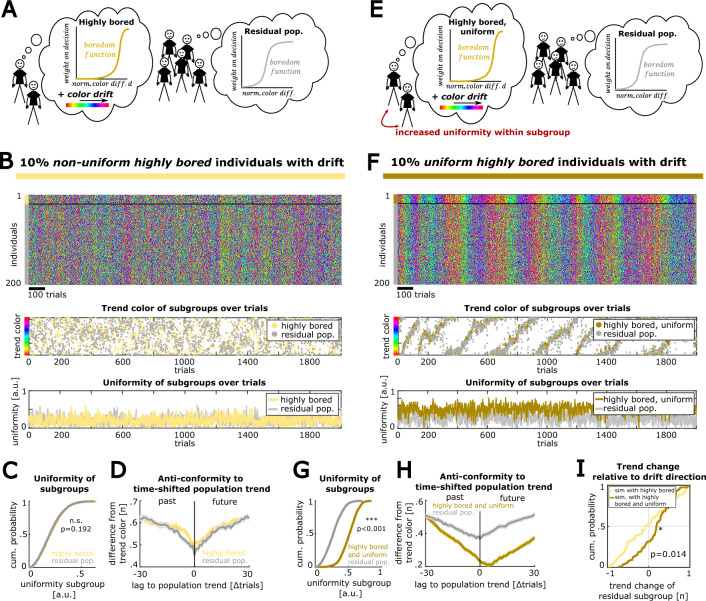
Uniformly acting highly bored individuals can drive population trends: (**A**) Schematic of a population with highly bored individuals that possess a systematic drift. (**B**) Simulation of this condition (model parameters: n_i_ = 200 individuals, 10% = 20 highly bored individuals with x_0_ = 0.9, boredom of residual population x_0_ = 0.75, memory size m = 10). Top: single individual color choices. Mid: Subpopulations’ trend colors over trials. Drift in highly bored individuals does not lead to a systematic drift in the subpopulations’ trend color. Bottom: Uniformity of both subpopulations over trials. (**C**) The uniformity between subgroups shows no significant difference (n = 2000 trials, p-value refers to Wilcoxon signed-rank test). (**D**) Mean absolute difference of the subpopulations’ trend colors to the full population trend colors, shifted over trials (n = 2000 trials). Shaded areas indicate the SEM (n = 2000 trials). (**E**) Schematic of a population, where the highly bored individuals possess a drift and have a bias towards choosing uniformly within their subgroup (see “Methods” section). (**F**) Same as B but for the highly bored subpopulation that has a bias towards behaving uniformly. Here, the drift manifests in the highly bored subpopulation’s trend color and propagates to the residual individuals’ trend. (**G**) Distributions of uniformity for both subgroups confirm the systematic uniformity bias for the highly bored subgroup (n = 2000 trials, p-value refers to Wilcoxon signed-rank test). (**H**) Same as D, where the highly bored and uniform individuals predict population trends in the close future. (**I**) Comparison of the efficacy in propagating the drift of the highly bored subgroup to the residual subgroup. Distribution of the change in the residual subgroup’s trend color at steps of 25 trials (positive values indicate a trend change in the direction of the drift, negative values indicate a trend change in the opposite direction of the drift, data from n_subset_ = 80 trials, the reported p-value refers to a Wilcoxon rank sum test).

In a next step, we tested the conditions in which a subgroup of highly bored individuals can influence upcoming population trends. Therefore, we created a modified version of our model, where a small fraction (10%) of individuals were endowed with high boredom and a systematic drift that was added to their color choice on each trial, implemented as a fixed additional rotation of their chosen color vector (Fig. 6A, “Methods” section). We then tested, if the residual individuals, that did not possess a drift, were affected by the drift of the highly bored individuals. Based on our previous observations, we hypothesized that the effectiveness of propagating the drift to the residual population depends on the uniformity of the highly bored subgroup.

We started with a combination of parameters that resulted in pronounced trends and a comparable level of uniformity in both subgroups (Fig. 6B,C, Wilcoxon signed rank test: n = 2000 trials, p = 0.192). However, the superimposed drift in the choices of the subpopulation of highly bored individuals did not become obvious in the collective behavior of the residual population and the difference between the respective subgroup trend and the time-shifted population trend was comparable (Fig. 6D). When enforcing a higher level of uniformity (see “Methods” section) in the subgroup of highly bored individuals (Fig. 6E,G, Wilcoxon signed rank test: n = 2000 trials, p = 2e−298), a systematic drift of their trend color became apparent that also propagated to the group of residual individuals (Fig. 6F). Comparing the difference between the respective subgroup trend and the time-shifted population trend revealed that highly bored individuals dominate future population trends and display avantgarde behavior (Fig. 6H). Overall, a subpopulation of uniform and highly bored individuals can propagate a systematic drift to the trend of the residual subpopulation (Fig. 6I, Wilcoxon rank sum test: n_subset_ = 80 trials with intervals of 25 trials, p = 0.014). Thus, our findings suggest that highly bored individuals can drive population trends and act as trendsetters of the future when behaving uniformly within their subgroup.

### Popularity amplifies the effect of highly bored individuals on a population

Based on the observation that trends in a highly uniform subgroup of individuals can propagate to the full population, we next explored a mechanism that enhances the impact of single individuals on the population. In particular, we implemented different degrees of *popularity* for some individuals in our model to test the effect of high popularity combined with different degrees of boredom on collective trend behavior.

**Figure 7:**
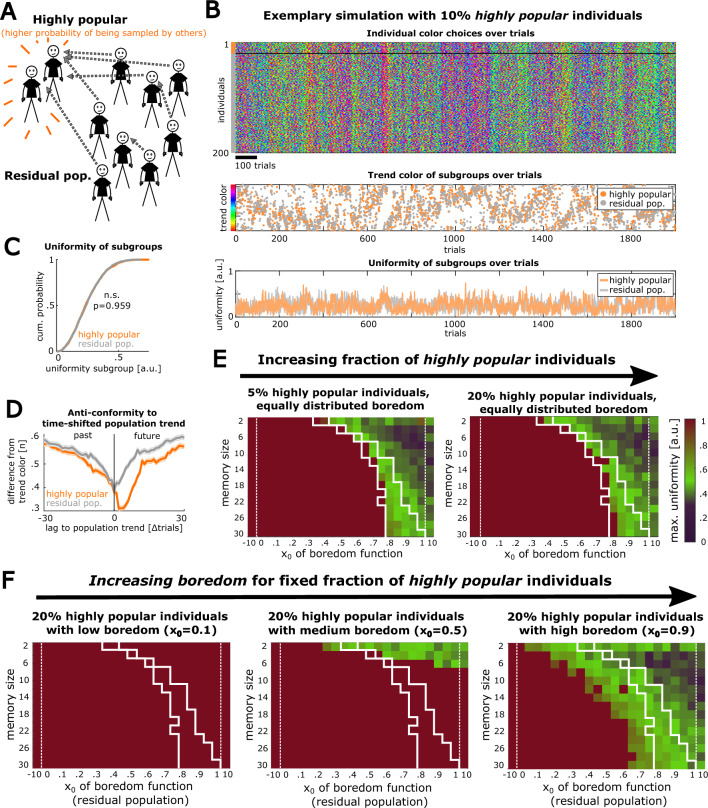
Higher popularity of highly bored individuals intensifies effects on collective trend dynamics. (**A**) Schematic illustrating that a defined subset of individuals from the population was constrained to have a high popularity (implemented as a likelihood to be sampled by others that was tenfold higher than for the residual individuals, see “Methods” section). The boredom function was homogenous across individuals. (**B**) Simulation of this condition (model parameters: n_i_ = 200 individuals, 10% = 20 highly popular individuals, memory size m = 10, boredom of population x_0_ = 0.75). Top: Single individual color choices. Mid: Subpopulations’ trend colors over trials. Bottom: Uniformity of both subpopulations over trials. (**C**) Uniformity between subgroups, showing no significant difference (n = 2000 trials, p-value refers to Wilcoxon signed-rank test). (**D**) Mean absolute difference of the subpopulations’ trend colors to the time-shifted full population trend (n = 2000 trials). The highly popular subgroup predicts trends in the close future. (**E**) Maximal uniformity of simulations across the parameter space with two increasing fractions of highly popular individuals. The white frames are reproduced from Fig. 4F for comparison of the parameter conditions that lead to well pronounced trends with intermediate uniformity in a homogenous population of agents. (**F**) Maximal uniformity over parameter space simulations with the same conditions as in the right panel of E, but with a constrained boredom parameter x_0_ for the highly popular fraction of individuals. We gradually increased the degree of boredom in highly popular individuals.

Starting with a population in which all individuals have the same boredom parameter x_0_, we defined a subfraction (10%) of individuals to have high popularity, implemented as a tenfold higher likelihood of being sampled by other individuals compared to residual, non-popular individuals (Fig. 7A, “Methods” section). We found that also a population with a highly popular subgroup can develop marked color trends that cycle over time (Fig. 7B). Interestingly, although the uniformity of the highly popular subgroup was similar to the residual subgroup (Fig. 7C, Wilcoxon signed rank test: n = 2000 trials, p = 0.959), the highly popular individuals were much stronger in affecting upcoming color trends, indicating a trendsetting effect (Fig. 7D).

We next simulated collective trend behavior over the model parameter space with increasing fractions of highly popular individuals. We found that highly popular individuals can establish color trends in parameter regimes where random choices were dominating in a population with homogenous popularity (Fig. 7E, Supplementary Fig. 4). Combining the aspects of popularity and boredom, we varied the degree of boredom for a fixed subpopulation of highly popular individuals (20%), independent from the residual population (Fig. 7F). We observed that highly popular individuals with low boredom lead to general convergence and disruption of trend sequences in the full parameter space which we explored. On the other hand, highly popular individuals that are strongly bored lead to reliable, non-convergent collective trend dynamics in many parts of the parameter space. In summary, this demonstrates that popularity and boredom can work together in controlling collective fashion behavior by intensifying trends and preventing convergence to monotony.

## Discussion

In this study, our approach to model fashion is determined by two key criteria: First, the appearance of trends, reflected by an overrepresentation of particular colors at a given time point, and second, the instability of such trends over time. Investigating examples of color trends in fashion magazines, we observed that one out of three tested fashion magazines showed significant fashion dynamics, varying in their rates of change, with periods of stability alternating with shorter periods of high trend change. We recapitulate these features in an agent-based model of collective fashion trend behavior, emerging from the interaction of individual agents endowed with boredom as a central behavioral feature. In our model, boredom plays an essential role to counterbalance a memory parameter and thereby preventing the population to converge to a stable fixed point with full uniformity among individuals. Furthermore, we manipulate boredom in a subgroup of the population and find that highly bored individuals show on average higher dispersion from the population trend, but specifically drive future population trends when acting coherently. High popularity, implemented as a higher probability of being sampled by other agents, can intensify this effect.

Most models of fashion behavior, including ours, distinguish intrinsic individual factors and extrinsic interactional factors in order to simulate ongoing fashion dynamics^24^. Intrinsic properties were often implemented as a categorization of individuals into conformists that copy the behavior of others, or anti-conformists acting as innovators that generate novel behaviors^25,26,32^. Other models allow individuals to switch between different states—either adopting a trend or leaving it—over the course of a fashion cycle^33^. However, these approaches did not formulate cognitive mechanisms that could underlie the individual propensity to follow or omit a trend. With our model, we fill this gap and propose a comprehensive computational process to model dynamic choice behavior based on individual experience. This bottom-up approach allows agents to continuously adjust their states based on current samples of the population trend, being either more conform with the trend or more against it. Thus, although different computational approaches to model fashion dynamics are certainly possible, our framework demonstrates that fashion can already emerge from a homogenous population of dynamically acting agents characterized by memory and boredom parameters that both have well-defined neurocognitive foundations^17,18,34–41^. Similar to agent-based models of other types of collective behavior^42,43^, our approach allows to generate hypotheses about the conditions that individuals need to meet in order to exhibit fashion dynamics, by specifically investigating the antagonistic roles of these two parameters on collective behavior^44^.

The individual memory of previously sampled colors from the population, determining the upcoming color choice of an agent, promotes homogeneity in the population, whereas boredom, implemented as a decreasing decision weight for sampled colors that are similar to the own choice, promotes heterogeneity. Manipulating both intrinsic individual parameters, we find that in particular boredom has crucial effects on collective fashion behavior: If collective boredom is high, the heterogeneity within a population at a given period is also high, even to the degree that any fashion trend can be disrupted. On the other side, if collective boredom is low, the population can converge to complete homogeneity, leading to a stagnancy of fashion trends. Thus, balanced levels of boredom are essential for the emergence of collective fashion behavior in our model. Besides boredom, the memory parameter of individual agents also affected collective fashion behavior in our model, in particular determining the speed by which color trends can change.

Our implementation of memory utilizes a moving time window for integrating past experiences, in line with classical concepts of statistical learning^45,46^. For the implementation of boredom, we rely on a parametric function to express the impact of an experience depending on its unpredictability^15^. Based on human studies that showed increased behavioral preference for novel stimuli^47^ and a dependence of choice behavior on the sensory information content^15^, we apply a sigmoid boredom function that assigns a high decision weight to samples strongly deviating from prior experiences. Other, more complex functions describing individual boredom susceptibility could also be used to model fashion behavior in the future. A neural correlate of the boredom function in the brain can be found in the representation of unpredictability in dopaminergic prediction error signals^48,49^ and patterns of neural activity coding for the uncertainty of a current state^50–52^. Together, our model shows that individual cognitive and mental abilities can affect fashion trend cycles within a population.

To address the external and interactional factors which contribute to collective fashion trends, we simplify the agents' sampling of others to a random process. Introducing heterogeneity in the sampling process by implementing a subpopulation of highly popular individuals, we find a strong effect on collective trend dynamics by broadly propagating their own choices, hence increasing the population’s uniformity. Sampling in more complex network structures can in the future be readily implemented in our model allowing a more detailed investigation of external, interactional factors of collective trends, such as varying information about the current collective state^53^.

In our model periods of relatively slow trend change alternate with periods of high trend change that are characterized by low uniformity in the population, suggesting a broad sampling of choice alternatives. Similar non-linearities in the change rates of collective behavior have been reported for opinion dynamics in humans^54,55^ as well as for collective movement in non-human species^56–58^. Here, the exchange of information between individuals in the population and their social coherence have been identified to effectively mediate these trend dynamics^54,55,59^, where the influenceability of a population is highest during periods of divergence^60^. Moreover, neural dynamics of cortical representations are similarly characterized by alternating phases of stability and abrupt intermittent changes^61,62^. This observation integrates in the concept of neural metastability^63^ suggesting a delicate interplay of conformity and segregation in neuronal processes, that together determine individual switches in behavior.

Together, these findings indicate that there may be critical periods in which upcoming trends can be influenced efficiently by promoting certain choice variants in the population. In our model, we investigated the role of a subgroup of highly bored individuals, that tend to be anti-conform with the current population trend. We observe that highly bored agents can drive population trends efficiently when making uniform, coherent choices. This aligns with other empirical observations^64^, suggesting that coherently acting, highly bored individuals can form an avantgarde in a population, influencing the trends of the future.

In our model, we simulate fashion trends in a circular space of behavioral alternatives given by a specific circular color spectrum. Despite mixed empirical evidence for color trends across different fashion magazines, we are able to account for aspects of non-random trend dynamics when projecting colors to a circular, perceptually even color subspace. However, a circular space is not appropriate for all behaviors that are affected by fashion trends. As such our model remains abstract and there may be multiple facets of fashion dynamics that will not be captured. For example, in our model the color space is evenly sampled over time, whereas we observed clear clusters of pale, reddish and dark trend colors in the data from fashion magazines. Moreover, as other factors, not covered in our model, might additionally affect fashion trends, our model does not attempt to explain collective fashions in all their details and hence needs to be interpreted non-exclusively. It rather proposes one agent-based solution with two elementary cognitive features that suffice to simulate trend dynamics and generate predictions about the role of individual features for collective behavior^44^. Hence, it is expected that the antagonistic principle of boredom and memory to shape collective trend behavior will translate also to other choice geometries and our model can provide a general framework to study fashion also in other contexts. For instance, boredom might constitute a relevant factor for information cascades in human opinion dynamics^59,65^, by affecting an individual’s affinity to exchange and communicate trend-related information with other individuals in a population^55^. This perspective is in line with previous investigations that characterized boredom as an internal signal to increase the information input to the brain^15,66^.

What is the empirical evidence that boredom may play a role in fashion? It is well established that boredom, describing either a transient mental state, or the individual’s general proneness to become bored can vary substantially among human individuals^4,67–70^. Furthermore, moderate boredom can have beneficial effects on individual behavior by enhancing exploration^13,14,16^, whereas high boredom is typically associated with dysfunctional behavior such as extensive risk seeking^6,71,72^. In line with this, previous research points out a link of boredom and anti-conformity-related constructs, such as innovativeness^73^ and creativity^13,74^ at an individual level. It therefore appears plausible that boredom may have an influence on the development of fashion trends.

Summarizing the observations of this study, our model leads to three main predictions: (i) The general level of boredom in a population affects the development of fashion trends. Thus, we expect that fashion markets, filled with individuals that are highly boredom prone, show faster and less coherent fashion cycles. (ii) Subgroups in the population can influence trends effectively when behaving coherently. Hence, a novel fashion variant should have the highest effect on future trends when being promoted in a coherent fraction of consumers during periods of changing population trends. (iii) Fashion trends can be highly affected by popular individuals with high boredom. Thus, popular persons with high boredom should be better predictors of upcoming fashion trends than popular persons with low boredom. We hope that with these predictions derived from our model, we set a starting point for further empirical studies which will be necessary to specifically unravel and validate the role of boredom on individuals decision-making and its impact on collective trends.

Here, we propose a generative and quantitative model that captures aspects of collective fashion behavior, with memory and boredom as main parameters. We demonstrate that in our model boredom can drive collective trend dynamics and shape future fashion trends, thus ensuring innovation and preventing stagnancy.

## Methods

All analyses and simulation were conducted using the software MATLAB®, including the MATLAB® statistics and machine learning toolbox (The Mathworks Inc., Natick, Massachusetts, USA, version R2022a).

### Analysis of trend colors in fashion magazines

In order to empirically test for color trends in popular fashion magazines, we extracted the dominant clothing color from the covers of three magazines that appear in a monthly rhythm: *Cosmopolitan, Vogue* and *Harper’s Bazaar*. We first acquired an image dataset with the monthly magazine covers for the time period from January 2000 until April 2022. Images were collected by systematic research from the websites www.pinterest.com, www.images.google.com and www.archive.vogue.com. Next, we extracted the dominant *RGB* color code with a custom written MATLAB® software as the mean from a small representative area of interest on the respective cover image. These raw trend colors were then transformed into *CIELAB* colors with the built-in function rgb2lab. The CIELAB space provides a perceptually even space to characterize colors and their distances relative to each other^30^. For all trend colors in CIELAB space, pooled over magazines, we then conducted a principal component analysis to project colors onto the plane of the first two principal components, that explained the most variance in the color data (85.6%). Based on this projection plane, we then represented and processed colors as the clockwise angle between a fixed *reference vector* $$\left[\begin{array}{c} 0\\ 1\end{array}\right]$$ and the two-dimensional *color vector* that connects the origin with the respective color coordinate. This procedure resulted in a projection of trend colors into a confined circular subspace of the CIELAB space (see Supplementary Fig. 1A, Fig. 1A,B).

#### Assessing the color trend over time

In order to analyze trends in circular color space over time, each color vector, connecting the origin and the coordinate of a given color in the principal component plane explaining most variance in the CIELAB space, was normalized to a length of 1. For the analyses of color trends in fashion magazines, the moving sum vector of the color vectors in a time bin of 8 months per magazine was computed and displayed. In our framework of color vectors this moving sum operation corresponds to building a moving average.

#### Assessing trend turnover

We sought to find a measure of how strongly color trends vary over time. For this purpose, we computed the speed of trend changes as the absolute angular step-by-step difference of the trend color over time (first derivative of trend color sequence). In a first step, this yielded absolute difference values in the range from 0 to +1. However, as we operationalized trend colors to be arranged in a circular space, we then adjusted the difference values to the color circle, where the highest possible difference (diametral vectors to opposite sides of the color circle) corresponds to an absolute difference angle of 1π (= 180°, Fig. 2A). Therefore, difference values > 0.5 were transformed by the operation 1-diff and later normalized by multiplying with a factor of 2, in order to obtain values in the range of 0 to + 1π for the trend change per trial. This procedure of processing empirical trend color changes was equivalent to our modeling procedures in the following steps (see “Methods” section below).

In order to test the statistical significance of the empirical color trend dynamics, we compared the distribution of trend changes from a given fashion magazine with the distribution of trend changes obtained after shuffling the respective trend color sequence randomly across time points (smoothening with the moving average was always applied after shuffling). A smaller average trend change of an empirical dataset compared to a shuffled one indicates non-random and temporally inter-dependent fashion dynamics. Applying a one-tailed Wilcoxon rank sum test to compare the empirical and shuffled condition against each other revealed statistical significance only for the trend dynamic of the Cosmopolitan (n = 267 trend changes, p = 0.007, Fig. 1F), but not for the Vogue (n = 267 trend changes, p = 0.895, Supplementary Fig. 1D) and Harper’s Bazaar (n = 267 trend changes, p = 0.287, Supplementary Fig. 1E). The observed non-random trend dynamics in the Cosmopolitan were robust against a Bonferroni-correction for multiple testing (corrected p = 0.021).

#### Coherence of color trends across fashion magazines

In order to test the synchrony of color trends in the three different fashion magazines Cosmopolitan, Vogue and Harper’s Bazaar, we computed the uniformity of the mean trend color vector over magazines and compared it to the uniformity of trend color sequences that were shuffled over time. In analogy to our later model analyses, we computed uniformity across magazines on a given month as the length of the summed color vector over magazines ($\overrightarrow{y},$ i_mag_ = [1,2,3]), normalized by the number of magazines (n_mag_ = 3): $uniformity=\frac{||\sum_{{i}_{mag}=1}^{3}{\overrightarrow{y}}_{{i}_{mag}}||}{3}$ (Supplementary Fig. 1B). Comparing the uniformity of empirical and shuffled trend color sequences did not reveal a significant difference, indicating independent fashion dynamics across magazines (Supplementary Fig. 1C, Wilcoxon rank sum test: n = 268 trend colors, p = 0.296).

### Detailed description and implementation of the boredom-fashion model

We propose an agent-based model to simulate choice behavior of a population of individuals over a sequence of points in time. We apply the model to fashion behavior, namely the repeated choice for colors from a circular space (Fig. 2A). Despite this focus on fashion and color, the model’s principles are generalizable to other domains of behavior which comprise a circular space of alternatives.

Specifically, we model the fashion choice behavior of each individual *i* within a population of *n*_*i*_ individuals over the sequence of trials *t* = 1,2,3,…,*n*_*t*_ (discrete time). For this modeling of fashion, each individual agent on each trial forms a choice of a clothing color based on memorized colors that it has sampled in the past. Then, the agent interacts randomly with another agent from the population, sampling and memorizing its current clothing color. The impact of each sampled color on following choices is determined by the degree of boredom it elicits (expressed as similarity to the own color choice; a visualization of this analogy is provided in Fig. 2B).

Thus, single-agent behavior on each trial comprises two processes: first, an individual *output* (the current choice for one color as the average of previously sampled colors), and second, reception of an *input* (the sampled clothing color of a random other individual from the population) that will affect the output on the next trial. For these processes, each individual is characterized by two parameters: (i) a memory capacity *m* that defines the number of previously sampled colors which affect the current choice (describing how many past samples are integrated into the current color choice), and (ii) a boredom term *w(d)* that weights a currently sampled input color by the dissimilarity *d* from the own current color (describing how susceptible an individual is to be bored by a given color difference). The variant space for the individual decision (different colors) is a circular color space in which diametral colors have the highest dissimilarity. In this framework, a specific color can be expressed as the angle $\theta$ between a vector $\overrightarrow{x}$ representing the color and the fixed reference vector $$\left[\begin{array}{c} 0\\ 1\end{array}\right]$$ on the unit circle (Fig. 2A):

$$\theta ={\mathrm{cos}}^{-1}\left(\frac{\overrightarrow{x}.\left[\begin{array}{c}0\\ 1 \end{array}\right]}{\left|\overrightarrow{x}\right|}\right) .$$

Accordingly, color vectors are used to represent chosen and sampled clothing colors in the model.

The model algorithm comprises the following computations on each trial.

*Step 0* The memories of all agents are initialized with random color vectors with length = 1, hence setting a random starting point for following trials.

*Step 1* An individual agent’s color choice $\overrightarrow{y}$ on the current trial *t* is expressed as the sum of all color vectors $\overrightarrow{{x}_{j}}$ in this agent’s memory:

$$\overrightarrow{y} = \sum_{j=1}^{m}\overrightarrow{{x}_{j}},$$

where *j* = 1,2,…,*m* is indexing the previous *m* trials *t-m, t-m* + 1,…, *t*−1. (for the first trials referring to the random initialization) (Fig. 2B-1,2).

*Step 2* The respective agent *i* interacts with a random other agent i* and samples this other agent’s current color vector $\overrightarrow{{z}_{i}}=\overrightarrow{{y}_{i*}}$ as input (Fig. 2B-3). The sampled input color vector $\overrightarrow{z}$ of the respective individual is then weighted by a *boredom function* $w(d)$, relative to the individual’s own current color $\overrightarrow{y}$(Fig. 2B-4).

*Step 3* The weighted sampled input color vector $w\left(d\right)\overrightarrow{z}$ is then stored in the agent’s memory list of vectors $\overrightarrow{{x}_{j}}$ at index *j* = 1, where all other samples from the previous trials are shifted by one index, dropping out the color vector which is furthest in the past (Fig. 2B-5).

The steps 1–3 are iteratively repeated for all individuals in the population, and in a hierarchically higher loop also for all trials of the simulation. Since the presented output color $\overrightarrow{y}$ on the next trial is computed as the sum over all sampled color vectors in the memory, those colors that yielded a higher difference from the chosen color will have a higher decision weight (i.e., a greater length) and hence will have a stronger impact on subsequent choices.

#### Boredom function

The boredom function serves the implementation of boredom in the modeled agents, characterized by an aversion towards monotony^4^. It is designed in a way that monotonous samples (i.e. with a low difference between output and input color) have only a low impact (weight) on an agent’s future decisions, whereas samples with much novelty (i.e. high difference between output and input color) have a high impact on an individual’s future decisions. Importantly, this implementation of boredom does not direct an agent’s choice to any specific attractor, which would be typical for curiosity-based frameworks^31,75,76^. It rather acts as a non-directed ‘push factor’ that drives an agent’s choices away from its prior experience^15^. The degree of monotony vs. novelty of a given sampled input color is computed as the angle $\delta$ between the chosen output color vector $\overrightarrow{y}$ and the sampled input color vector $\overrightarrow{z}$ on the current trial

$$\delta ={\mathrm{cos}}^{-1}\left(\frac{\overrightarrow{y}.\overrightarrow{z}}{\left|\overrightarrow{y}\right| \left|\overrightarrow{z}\right|}\right).$$

This angle $\delta$ is then normalized to the maximally possible difference angle between diametral color vectors ($\pi$), yielding the difference score *d*, ranging from 0 to 1 in units of π (see Fig. 2B-4)

$$d=\frac{\delta }{\pi }.$$

For the boredom function, we apply an increasing sigmoid. This function *w*(*d*) maps a given value of difference *d* between sampled input and own choice onto a decision weight *w*, expressing how much the current sample will affect future decisions. The sigmoid function relies on the empirical observation that boredom-related aversion increases non-linearly with the degree of monotony in a situation^15^. The sigmoid boredom function is formulated as

$$w\left(d\right)=\frac{1}{1+\mathrm{exp}\left(-20 (d-{x}_{0})\right)},$$

where *x*_0_ indicates the shift of the function along the abscissa reflecting *d*. Here, high x_0_ values correspond to a right-shifted sigmoid, reflecting high boredom proneness (i.e., high decision weights are only reached for samples that contain much novelty and hence have a high *d* value), whereas low x_0_ values would correspond to a left-shifted sigmoid, reflecting low boredom proneness (i.e., high decision weights are obtained already for samples with little novelty, hence having a low *d* value). The maximum and skewness of the function are constrained in order to specifically test the effect of shifting the boredom function horizontally in order to simulate different levels of boredom.

The respective decision weight *w(d)* is then used as a scaling factor for the length of the sampled input vector $\overrightarrow{z}$ (see above).

### Computing uniformity

The uniformity within the population on a given trial is computed as the normalized length of the summed color vector of all individuals *i* = 1,2,…,*n*_*i*_. Specifically, for a given trial, all presented color vectors $\overrightarrow{{y}_{i}}$ are scaled to have a length = 1. Then, uniformity is calculated as

$$uniformity=\frac{||\sum_{i=1}^{{n}_{i}}{\overrightarrow{y}}_{i}||}{{n}_{i}}.$$

In order to estimate the degree of uniformity that we observed in a model simulation, we further established a threshold that describes the uniformity that can be expected by chance. For this purpose, we created a distribution over 10,000 iterations where we instantiated random color vectors for all simulated individuals (n_i_ = 200) and then computed the uniformity over those random vectors. This led to a distribution of 10,000 uniformity scores. We then chose the uniformity threshold according to the 99th percentile of this distribution (uniformity_threshold_ = 0.153).

### Introducing heterogeneity in the population and probing the parameter space

In order to investigate the effects of highly bored individuals and highly popular individuals on the population trend dynamics, we explored model simulations in the space of different parameter combinations.

First, we simulated a homogenous population. Here, we constrained the population size (n_i_ = 200 individuals) and the number of trials (n_t_ = 2000), while systematically altering the population’s *memory size m* and horizontal shift of the *boredom function x*_0_. The maximal uniformity of each simulation was used as main read-out for the trend dynamic of the respective parameter combination. This maximal value reflects how reliably a population develops color trends. The maximum has the advantage over average measures to capture a convergence of trend colors, even if this convergence might occur only at the end of a simulation—a scenario where the average over trials would be overshadowed by the majority of trials before convergence, hence underestimating the uniformity of the population.

In addition, we computed the mean absolute speed of change of the population’s trend color (in units of pi) in order to obtain a measure of general turnover of color trends. In the domain of fashion behavior, this measure could reflect how much a population on average cycles their fashion items.

#### Changing the fraction of highly bored individuals

Starting from a condition where all individuals have equivalent attributes, we systematically increased the fraction of highly bored individuals, implemented as individuals whose boredom function was characterized by a shift of x_0_ = 0.9. For each given fraction of highly bored individuals, we simulated choice behavior in the same parameter space as for the naïve model (all individuals and seeds are equivalent), by changing the memory size and x_0_ parameter of the residual population. The difference between each subpopulation’s trend color and the full population trend color was computed independently for both subgroups, where the residual subgroup was constrained to a random subsample of the same size as the highly bored subgroup. Furthermore, we computed uniformity for both subgroups.

In order to compare how the trend uniformity in a heterogeneous population with a defined fraction of highly bored agents relates to the uniformity in a homogenous population with comparable average boredom, we selected all fields from the parameter space matrices under both conditions that had equivalent mean boredom parameters (*x*_0_) and the same memory (*m*). For instance, the field in the parameter space matrix of the heterogenous population with $m=12$, ${x}_{0\_residual}=0.65$ and 20% highly bored individuals with ${x}_{0\_highBoredom }=0.9$ displayed an average boredom parameter of ${x}_{0\_mean}=0.8\times 0.65+0.2\times 0.9=0.7$ and hence was compared to the field in the parameter space matrix of the homogenous population with $m=12$ and ${x}_{0}=0.7$. For all equivalent parameter combinations between the two matrices, we built the differences and compared them in a Wilcoxon rank sum test against a median of zero.

#### Quantifying the relationship of subgroup trends and the full population trend

We aimed to test the effect of a specific subgroup in the population (e.g., a highly bored subpopulation) on the trend of the full population. Therefore, we quantified the trend color sequence over all trials for the respective subgroup and for the full population. Then, we computed the mean difference between the subgroup’s trend color sequence with the trend color sequence of the full population (mean difference over n = 2000 trials). We repeated this procedure with permuted versions of the trend sequence of the full population, that we shifted in time by a specific number of trials (lag ranging from − 30 to + 30 trials, covering time-shifts into the past and future). By finding the difference minimum over time-shifts, this analysis allowed an investigation of the time period in the full population trend sequence that was most similar to the subgroup trend on a reference time point.

#### Quantifying the impact of single individuals on changes in the population trend

In order to assess the impact of a single simulated agent on changes in the population trend, we computed an *impact index* based on how often a given individual was sampled by others and based on the weight that this sampling had on other individuals’ decisions. Specifically, the impact index for an agent *i*, that chose the color vector $\overrightarrow{y}$ on a given trial *t* was computed as

$$impact \, inde{x}_{i,t}=\sum_{j=1}^{{n}_{j}}{w}_{j}\left({d}_{y}\right)\times {\mathrm{cos}}^{-1}\left(\frac{\overrightarrow{y}.\overrightarrow{{s}_{t+1}}}{\left|\overrightarrow{y}\right| \left|\overrightarrow{{s}_{t+1}}\right|}\right),$$

where, ${n}_{j}$ indicates the number of other individuals that sampled agent *i*, ${w}_{j}\left({d}_{y}\right)$ denotes the decision weight that each of these other individuals attributed to the sample of $\overrightarrow{y}$ and $\overrightarrow{{s}_{t+1}}$ reflects the full population’s trend color (summed color vector) on the upcoming trial *t* + 1. This metric is expressing the aggregate change of the trend color that an individual induces in the population with its choice on a given time point. A high impact index hence reflects a strong effect of a given individual on changing the population trend, whereas a low value reflects a low effect on changing the population trend.

#### Testing the impact of a systematic choice drift in a highly bored subpopulation

We aimed to test how effective a subgroup of highly bored individuals can affect and potentially drive color trends in the residual population. Therefore, we modified our model such that the color choice of each individual from the highly bored subgroup was unidirectionally drifting in the color space by a fixed value. This was implemented by rotating each high-boredom individual’s color vector on each trial by 17° clockwise around the origin. This rotation was added to the basic sampling and weighting of other individuals’ colors, hence leaving the choice principles of the model intact. We then tested in how far the systematic drift of highly bored individuals propagated and induced changes in the subgroup of residual individuals that did not possess any color drift. For this purpose, we compared the change between residual trend colors at intervals of 25 trials. This change was expressed in units of π as described above for the angle between color vectors, and had a positive value if it was matching the direction of drift in the highly bored subgroup, whereas a negative value indicated an oppositional direction compared to the drift in the highly bored subgroup. Two conditions were simulated: first, a subgroup of highly bored individuals with drift, and second, a subgroup of highly bored and uniform individuals with drift. Here, the bias towards high uniformity was implemented by adding the summed color vector, computed over all highly bored individuals, to these individuals’ color choices on each trial. In order to ensure a fixed impact on each trial, this added subpopulation trend vector was weighted by a factor that scaled it to 40% of the current color vector, computed over all memorized items.

#### Changing the fraction of highly popular individuals

High popularity was implemented by changing the probability for a given individual of being sampled by others (meaning that other individuals on a given trial sample this agent’s current color). The probability of being sampled by others for highly popular individual*s* was set to be tenfold the probability of being sampled for the non-popular, residual individuals.

In order to test the conformity of individuals with the population trend we conducted an analogous analysis as described above, comparing the highly popular and residual subgroup trends against the full population trend.

#### Changing the degree of boredom in highly popular individuals

In order to test the effect of high boredom in individuals with high popularity, we combined both previous approaches. For specific subpopulations of highly popular individuals, we systematically varied the boredom parameter x_0_. The memory size and boredom attribute of the residual population were kept constant for a given simulation, where different simulations were conducted to systematically explore the parameter space, in line with the analyses mentioned above.

### Statistical tests

We followed a combination of approaches in order to test the statistical reliability of our findings. All procedures are in detail explained in the respective section of the main text. The correlation between uniformity within a population and the speed of trend color changes was probed using Pearson correlation. The relationship between individuals’ uniformity and their impact on population trends was quantified using Spearman correlation. In order to test for group differences, we conducted Wilcoxon signed rank tests or Wilcoxon rank sum tests.

### Supplementary Information

Supplementary Figures.

## Data Availability

The data and model code of this study is available from the corresponding authors upon reasonable request.
