# Supplementary material for: Modeling fashion as an emergent collective behavior of bored individuals
Source: Sci Rep. 2023 Nov 22;13:20480. doi: 10.1038/s41598-023-47749-7 (PMC10665449; doi:10.1038/s41598-023-47749-7)
Supplement: Supplementary file 1 — Supplementary Figures. [file 41598_2023_47749_MOESM1_ESM.pdf]

Modeling fashion as an emergent collective behavior of bored individuals

Johannes P.-H. Seiler[1], Simon Rumpel[1]

[1] Institute of Physiology, University Medical Center of the Johannes Gutenberg University Mainz,
Hanns-Dieter-Hüsch-Weg 19, 55131 Mainz, Germany

Correspondence concerning this article should be addressed to Simon Rumpel or Johannes Seiler, Institute of Physiology, Focus Program Translational Neurosciences, University Medical Center, Johannes Gutenberg University-Mainz, Hanns-Dieter-Hüsch-Weg 19, 55131 Mainz, Germany. E-mail: sirumpel@uni-mainz.de; johseile@uni-mainz.de

Additional information

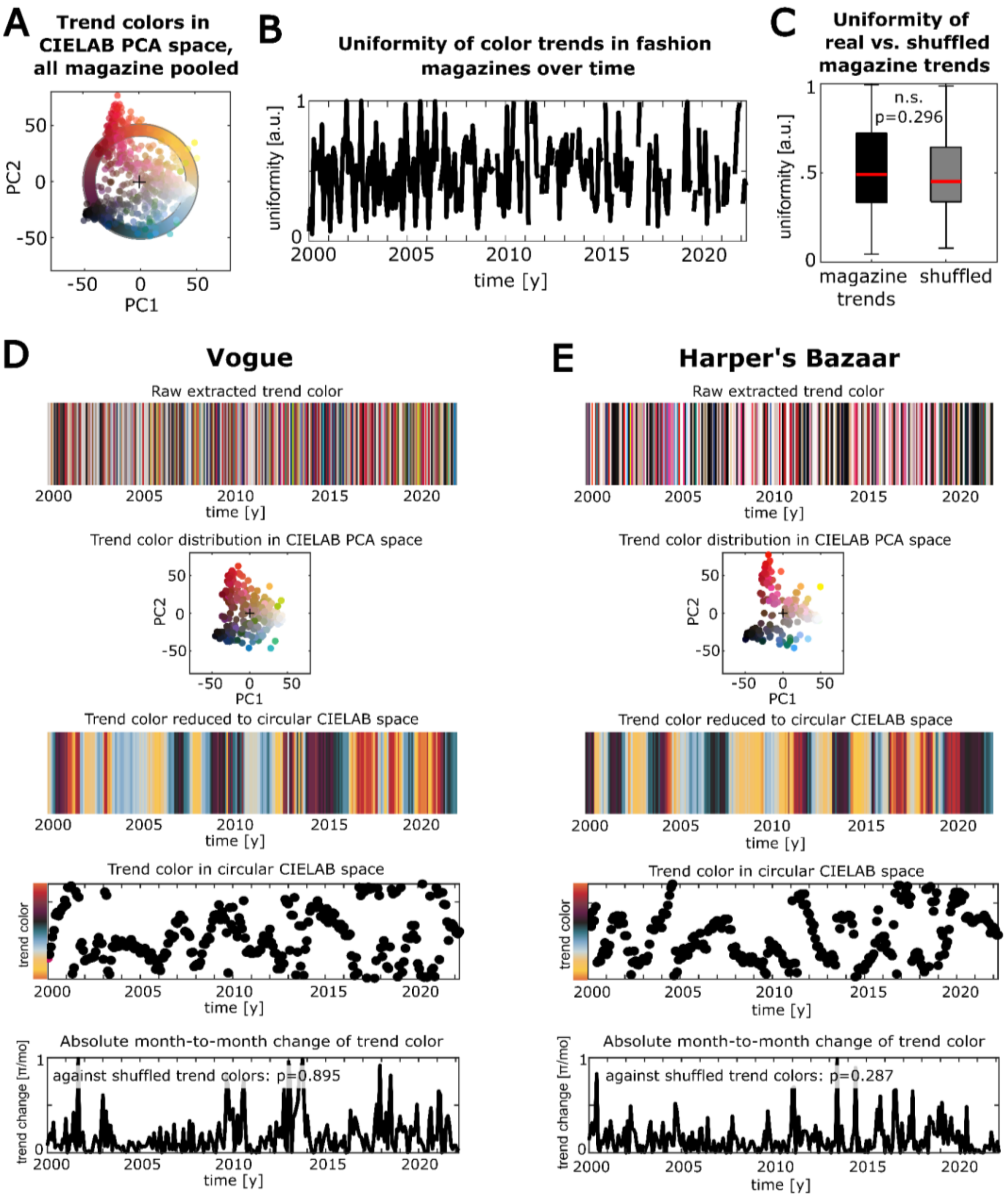

Supplementary Figure 1 –Color trends and their coherence across different fashion magazines: (**A**) Pooled trend colors of all fashion magazines (n=804 trend colors from Cosmopolitan, Vogue and Harper's Bazaar) projected into the 2-dimensional plain that explains most variance of all trend colors in CIELAB space. This plain is further used to obtain a circular projection of all trend colors (see color wheel in the background). (**B**) As a measure of coherence between the trend colors from the three fashion magazines, we computed the uniformity of color vectors for each month (see Methods). (**C**) The coherence of the three magazine trends does not differ significantly from trend colors randomly shuffled over time, indicating no compelling synchrony of color trends across fashion magazines. (**D+E**) Detailed analysis of color trends in the fashion magazines *Vogue* and *Harper's Bazaar*. First panel from the top: Trend colors in RGB color space. Second panel from the top: Trend colors projected into the plain that explains most variance of all trend colors in CIELAB space. Third panel from the top: Trend color sequence after projection onto the circular subspace in the plain described above. Fourth panel from the top: Time-shifted sum over color vectors in a moving bin of 8 months over time. Fifth panel from the top: Month-to-month change between the trend colors over time (absolute first derivative from the panel above).

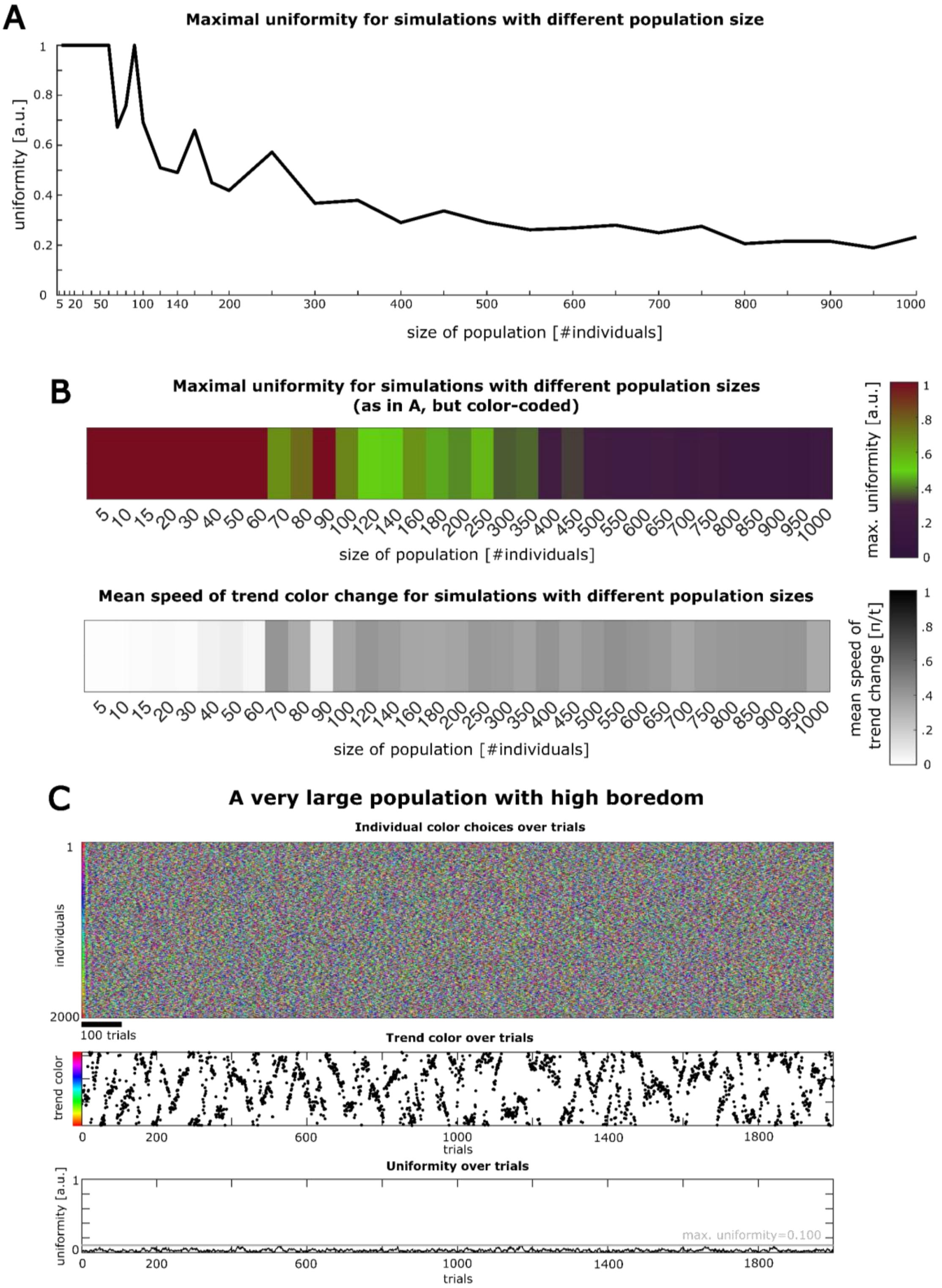

Supplementary Figure 2 – Population size affects the simulated collective trend behavior: (**A**) Maximal uniformity of different simulations (memory size m=12, x_0=0.7), conducted with varying population sizes. Small populations carry a greater risk of converging than large populations. Increasing the population size further than approximately 200 individuals has only a minor effect on collective trend behavior compared to increasing the population size in the range from 5 to 200. (**B**) Upper panel: Same as in A, but coded with the colormap that is also used in other parameter space explorations (Figures 4,5,7). Red color indicates a convergence, dark purple indicates random choices and green indicates reliable population trends. The best-developed trend dynamics can be observed in intermediately sized populations. Lower panel: Absolute difference in trend color change for the same simulations as in A and the upper panel of B. Dark color indicates a high speed of change in the

color trends of a population. Increasing the population size further after an intermediate level of approximately 100 individuals does not relevantly affect the overall speed of trend color change. (**C**) Simulated color trend dynamics for a very large population (n_i=2000 individuals) with high boredom proneness (x_0=0.9). Upper panel: Individual color choices over trials. Individuals from the population show random and unsynchronized color choices without the emergence of trends. Middle panel: Trend color of the population over time. Lower panel: Uniformity of the population over trials. The solid horizontal line indicates the maximal uniformity, being very low at chance level indicating random choice behavior in the population.

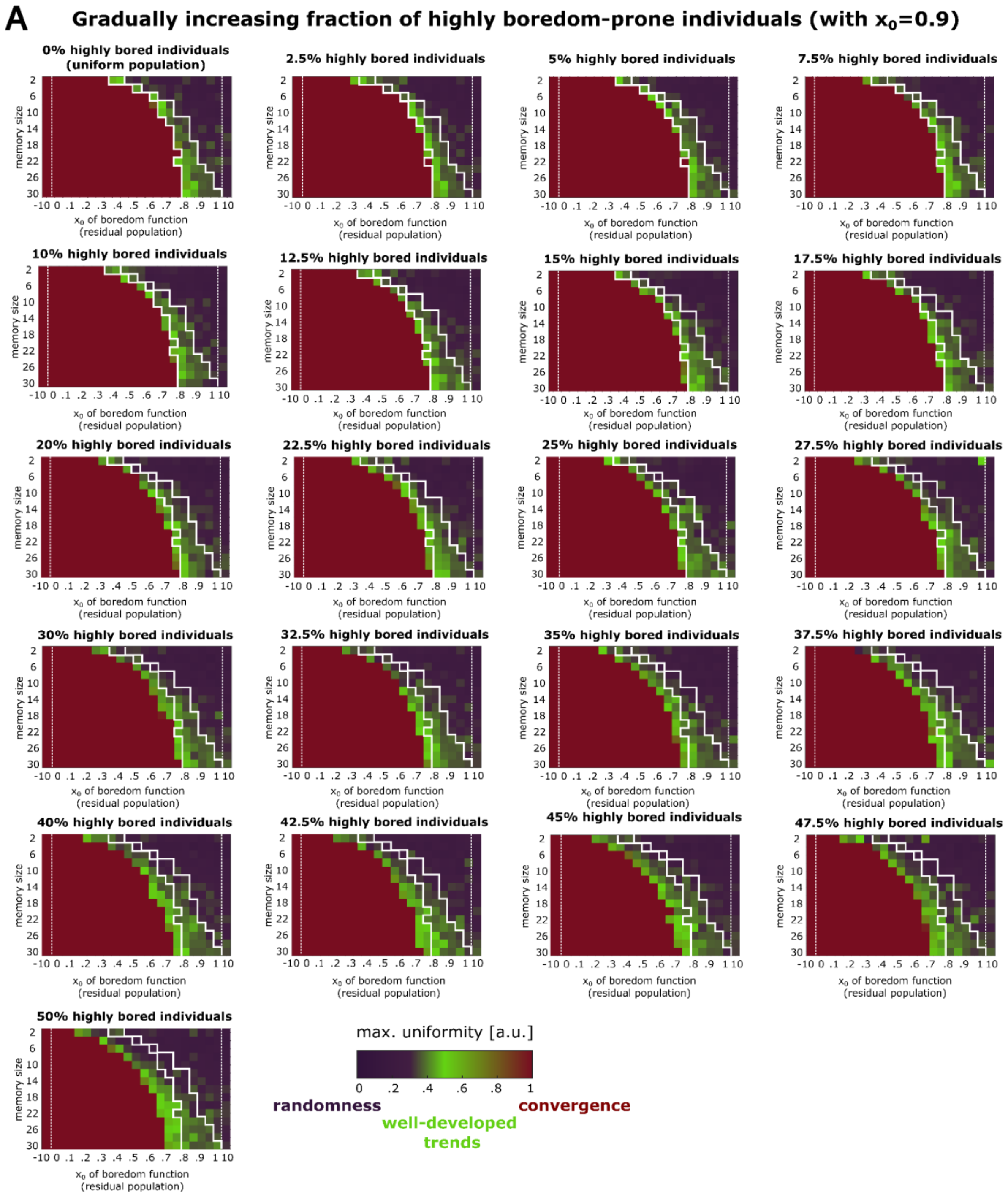

Supplementary Figure 3 – Extension of simulations in parameter space with gradually increasing fractions of highly bored individuals: (**A**) Maximal uniformity of the simulations across the parameters space with gradually increasing fractions of highly bored individuals. The white lines frame the area which showed well-developed collective trends in the homogenous condition with a uniform population (Figure 4F).

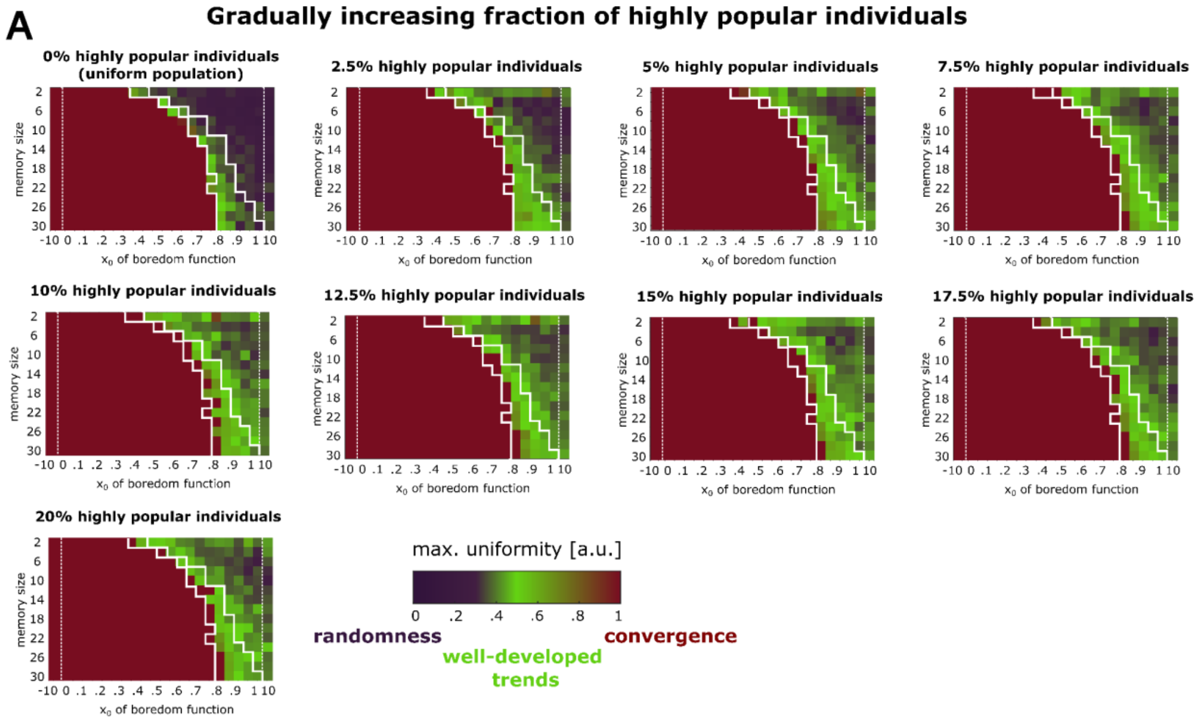

Supplementary Figure 4 – Extension of simulations in parameter space with gradually increasing fractions of highly popular individuals: (**A**) Maximal uniformity of the simulations across the parameters space with gradually increasing fractions of highly popular individuals. The white lines frame the area which showed well-developed collective trends in the homogenous condition with a uniform population (Figure 4F).
